# Hidden in plain sight: how to look behind the veil of cocaine-induced vasculitis

**DOI:** 10.1093/rap/rkad047

**Published:** 2023-05-05

**Authors:** Sander I van Leuven

**Affiliations:** Department of Rheumatology, Radboud University Medical Center, Nijmegen, The Netherlands

**This editorial refers to ‘Cocaine-induced granulomatosis with polyangiitis—an under-recognized condition’, by Gill *et al.*https://doi.org/10.1093/rap/rkad027.**

Cocaine is an illegal drug derived from the leaves of the coca plant native to South America. According to the latest report of the United Nations Office on Drugs and Crime, the area under coca bush cultivation in 2020 was roughly equivalent to an area three times the size of New York City. Global cocaine production has now more than doubled since 2014. The number of people who use cocaine has increased concordantly to almost 22 million or 0.4% of the global population aged 15–64 years [1]. The majority of seized cocaine is laced with the antihelmintic drug levamisole to increase profit, because it is not detected by standard purity kits and because it might potentiate the euphoric effects of cocaine by further increasing brain dopamine levels [2].

Cocaine can be used orally, intranasally, intravenously or by inhalation and can cause various deleterious effects on different organ systems (Fig. 1). The use of (adulterated) cocaine can trigger a pseudovasculitis presentation closely mimicking true idiopathic ANCA-associated vasculitis (AAV) [3, 4]. It is a clinical challenge for rheumatologists, nephrologists, pulmonologists, dermatologists and ENT specialists to differentiate a cocaine-induced vasculitis from AAV. In this issue of *Rheumatology Advances in Practice*, Gill *et al.* [5] were able to carry out retrospective analysis of a large number of patients who presented during a 5-year period in tertiary centres in London and Birmingham. In total, 42 patients with severe nasal damage mimicking AAV were included who disclosed cocaine use or had positive urine toxicology. This case series highlights several important issues with regard to epidemiology, diagnosis and treatment of cocaine-induced vasculitis.

**Figure 1.**
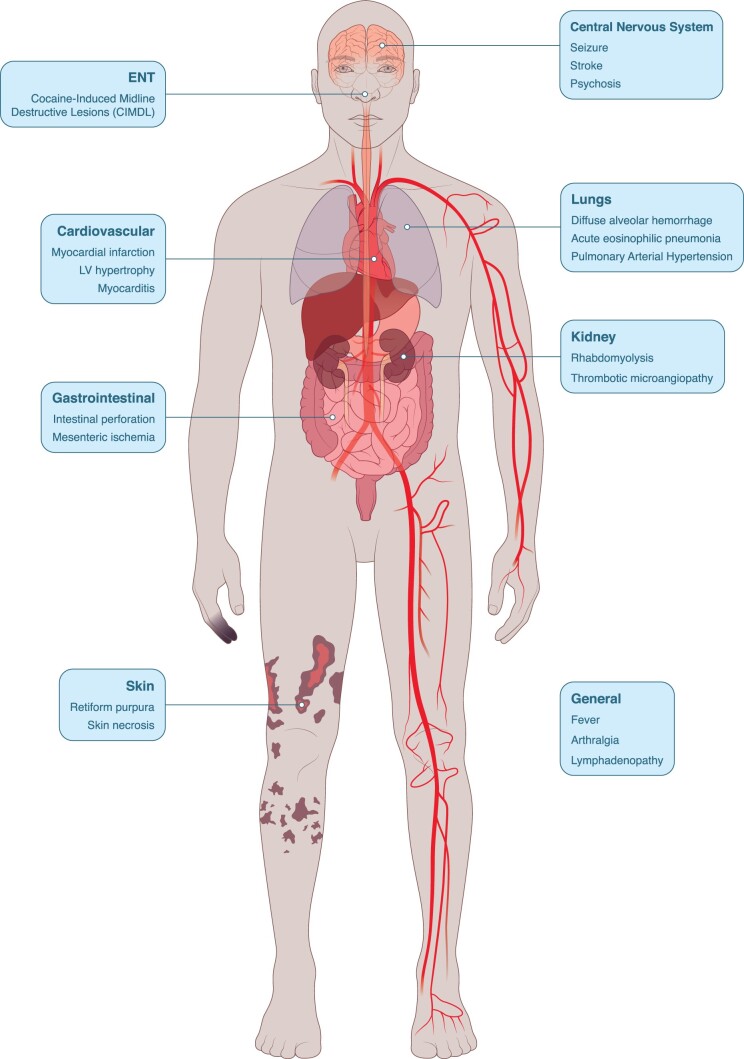
The use of cocaine is associated with various deleterious effects on different organ systems

As cocaine use continues to increase globally, cocaine-induced vasculitis is becoming more prevalent. This is perhaps shown indirectly by the observation that the number of included patients in published case series is growing. Granulomatosis with polyangiitis and microscopic polyangiitis affect men and women equally, which was also the case in this study on cocaine-induced vasculitis [5]. A higher prevalence in men has been reported previously [2], but it should be noted that men are more likely to use cocaine [1]. The mean age of patients with cocaine-induced vasculitis, however, was ∼20 years younger than that of AAV patients [5].

Although a thorough drug history is of major importance, this will not always suffice. In the Birmingham clinic, urine toxicology was routinely requested from patients. This revealed that in almost one-third of cases, patients would have been misdiagnosed without toxicology. Indeed, 8 of the 29 patients who were referred to the Birmingham centre had been diagnosed previously with AAV. The correlation between self-reported cocaine use and the results of urine assays is known to be poor [6], and the present study clearly illustrates that in patients with a high index of suspicion (e.g. isolated nasal involvement), toxicology should always be considered. Urine toxicology also revealed that half of the patients from Birmingham simultaneously used other drugs, which might cause additional symptoms and which can further complicate the diagnostic process. It should also be noted that urine toxicology for cocaine metabolites typically reveals the use of cocaine only ≤5 days before testing.

Almost 90% of patients in the present study were ANCA positive, and similar to other studies, the pattern was frequently atypical. Dual positivity for antibodies against both MPO and PR3 has previously been suggested to be highly suggestive of a drug-induced aetiology [7]. The present study does not support this, and the authors correctly point out that dual positivity has also been reported in AAV. Indeed, the ANCA subtype is not specific for cocaine-induced nasal damage. Different patterns of ANCA testing might also be caused by the fact that cocaine is laced with different adulterants in different parts of the world. Anti-human neutrophil elastase (HNE) antibodies might help to distinguish cocaine-induced vasculitis from AAV, but the HNE assay is not widely available.

Histopathology can be of use for differentiating AAV from cocaine-induced vasculitis. In total, 28 patients underwent nasal or sinus biopsy. This showed small vessel vasculitis in two patients, and none of the biopsies showed granulomatous inflammation. Extravascular necrosis, microabscesses, granulomas and giant cells are hallmarks of granulomatosis with polyangiitis and are absent in cocaine-induced vasculitis. Vascular abnormalities mimicking vasculitis might not be helpful in distinguishing AAV from cocaine-induced vasculitis because they have also been demonstrated frequently in biopsy specimens of patients with cocaine-induced midline destructive lesions [8].

In the present study, systemic disease was less prevalent than previously reported [2–4], which might be attributable to the fact the majority of patients came via an ENT referral pathway, as the authors indicate. In line with previous case series, this study also clearly shows that cessation of cocaine use is pivotal in order to achieve clinical resolution. Immunosuppressive therapies (including CSs, rituximab and CYC) were unable to induce remission on a background of ongoing cocaine use [5]. There is no standard treatment for cocaine use disorders. Recognition of cocaine as the culprit represents a prime opportunity for counselling patients regarding the risks of continued use and making efforts to link them to addiction counselling with psychosocial interventions or contingency management programmes [9].

Increased global drug use, however, is not limited to the use of cocaine. Around 284 million people aged 15–64 years used drugs worldwide in 2020, a 26% increase over the previous decade. Young people are using more drugs, with higher use levels than the previous generation [1]. Consequently, health-care services will be confronted more often with the detrimental effects of illegal (combined) drug use. For instance, in addition to cocaine-induced vasculitis, there has recently been an alarming increase in the incidence of major thrombotic events in young adults associated with the use of nitrous oxide (laughing gas) as a recreational drug [10]. Continued efforts to inform the general public of the severe consequences of (novel) drugs are obviously very important. Simultaneously, increased awareness among the medical community and clinicians in particular is crucial to prevent under-recognition, misdiagnosis and inappropriate (immunosuppressive) treatments.

## Data Availability

No new data were presented in this editorial.
